# Double shrinkage transfer causal learning: An application to alzheimer’s disease

**DOI:** 10.1371/journal.pcbi.1014706

**Published:** 2026-08-24

**Authors:** Yunxin Shi, Lulu Pan, Yu Hu, Yongfu Yu, Guoyou Qin

**Affiliations:** Department of Biostatistics, Key Laboratory of Public Health Safety of Ministry of Education, NHC Key Laboratory for Health Technology Assessment, School of Public Health, Fudan University, Shanghai, China; CSIR-IHBT: Institute of Himalayan Bioresource Technology CSIR, INDIA

## Abstract

Amyloid-Beta 42 (ABeta42) is a key biomarker of cerebral amyloidosis in Alzheimer’s disease, and estimating its causal effect on subsequent cognitive outcomes is important for understanding disease progression in racial minority populations, such as African Americans. However, causal effect analyses in such populations are often challenged by limited sample sizes, leading to biased or unstable estimates when only target-population data are used. Existing transfer causal learning methods borrow information from a larger related source domain, but may be unstable when source-domain estimation is high-dimensional or when irrelevant covariates introduce noise into transferred nuisance models. We propose a double shrinkage transfer causal learning (DSTCL) estimator that regularizes both the initial source domain estimation and the source-target parameter differences, and then estimates causal effects using a doubly robust framework. We evaluated DSTCL in simulations under varying source sample sizes, inter-domain similarities, covariate dimensions, and other settings, and then applied it to the Alzheimer’s Disease Neuroimaging Initiative dataset using African American participants as the target domain and non-Hispanic White participants as the source domain. In simulations, DSTCL achieved small bias, low mean squared error, and stable confidence interval coverage, generally outperforming competing methods. In the ADNI application, lower baseline ABeta42, reflecting greater amyloid burden, was associated with worse 12-month cognitive performance among African American participants, with DSTCL producing the narrowest confidence interval among the methods compared. These findings suggest that DSTCL provides a practical framework for transfer causal inference in biomedical studies with limited target population.

## Author summary

African Americans continue to be included in relatively small numbers in many studies of Alzheimer’s disease. We sought to improve the investigation of disease-related biomarkers and later cognitive health in populations for which only limited data are available. Lower levels of amyloid-beta 42 in the fluid surrounding the brain and spinal cord generally reflect greater amyloid accumulation in the brain, a hallmark of Alzheimer’s disease. However, drawing reliable conclusions is difficult when a study includes few participants but many potentially relevant characteristics. We developed a statistical approach that learns useful information from a larger, related population while retaining important differences in the smaller population and reducing the influence of irrelevant information. In the simulations, our approach generally provided more accurate and stable results than existing methods. We then applied it to data from the Alzheimer’s Disease Neuroimaging Initiative, using African Americans as the target domain and non-Hispanic Whites as the larger source domain. Under assumptions needed for causal interpretation, our findings suggested that lower baseline amyloid-beta 42 was associated with worse cognitive performance after 12 months among African Americans. These findings suggest that our approach provides a practical framework for biomedical studies when target population data are limited.

## 1. Introduction

Alzheimer’s disease (AD) is a progressive neurodegenerative disorder characterized by a gradual decline in memory and cognitive function, with its prevalence increasing worldwide in parallel with population aging [1,2]. Cerebrospinal fluid (CSF) amyloid-Beta 42 (ABeta42) is a core biomarker of cerebral amyloidosis and plays a central role in characterizing AD severity and disease progression trajectories [3,4]. Previous studies have shown that amyloid burden and clinical progression patterns differ across racial groups, with African Americans experiencing a substantially higher disease burden than non-Hispanic Whites [5,6]. These disparities motivate a more precise characterization of the relationship between amyloid biomarkers and disease severity in African Americans, to inform population-specific understanding of disease evolution and potential intervention strategies. However, progress in addressing these questions is constrained by fundamental data limitations. Major AD cohorts exhibit substantial imbalance in population representation, largely driven by limited sample sizes of African American participants. For example, in the Alzheimer’s Disease Neuroimaging Initiative (ADNI), which includes data from 59 North American sites and several thousand participants, African Americans account for only about 10% of the total sample [7,8].

Meanwhile, growing evidence underscores the importance of integrating molecular-level data to better capture AD mechanisms and heterogeneity. Genome-wide DNA methylation (DNAm) profiles reflect systemic and brain-related pathological processes and are widely used as covariates to improve confounding control in etiologic analyses [9,10]. Epigenome-wide meta-analyses further demonstrate that AD-related DNAm changes are broadly shared across cohorts, with a limited number of locus-specific differences [11,12]. While incorporating DNAm into causal analyses can substantially improve confounding control, it also increases model complexity and places greater demands on stable estimation, particularly when the target population sample size is limited. In such a setting, methods that estimate all parameters using only target domain data often perform poorly: classical doubly robust estimators that rely on matrix inversion may fail numerically, while regularization or machine learning alternatives can produce substantially biased and unstable causal effect estimates [13–16].

Transfer learning offers a promising avenue to mitigate these limitations. Its central idea is to improve performance in a target dataset by borrowing information from external source data, and it has demonstrated substantial potential in applications such as drug discovery and metabolic mechanism studies [17–19]. Künzel et al. were among the first to systematically investigate how transfer learning can be used for causal effect estimation under a shared covariate space, and referred to this as the transfer causal learning (TCL) problem [19]. Building on this, Wei et al. proposed the $l_{\text{1}}$-TCL method [20], which obtains initial parameter estimates via maximum likelihood in the source domain and then uses target data to estimate parameter differences, thereby achieving transfer learning of nuisance parameters and plugging the resulting estimates directly into causal effect estimators. The $l_{\text{1}}$-TCL method typically assumes a high degree of similarity in covariate structures between the source and target domains. However, when a traditional maximum likelihood method is used in the source domain at the initial stage, the resulting estimates often retain nonzero effect estimates for covariates with little true relevance to the outcome, thereby transmitting instability into the estimation of target domain parameters, potentially amplifying estimation error and undermining the accuracy and stability of causal effect estimates.

To address this issue, we propose a double shrinkage transfer causal learning (DSTCL) method that improves upon existing TCL approaches by applying shrinkage both to the initial source domain estimation and to the source-target parameter differences, yielding more stable and reliable causal effect estimates. We conduct systematic simulation studies to evaluate the performance of DSTCL across a range of scenarios varying in source sample size, inter-domain similarity, and covariate dimension, and apply the method to the ADNI dataset to estimate and quantify the causal effect of baseline ABeta42 status on subsequent cognitive function among African American participants. Our findings not only enhance understanding of AD-related biomarkers and disease progression in the African American population, but also provide a transferable statistical framework for causal inference in studies of racial minority populations and other biomedical studies.

## 2. Materials and methods

### 2.1. Ethics Statement

This study used simulated data and de-identified secondary data obtained from the ADNI database (https://adni.loni.usc.edu/). Ethics approval was not applicable to the simulations because they did not involve human participants or animals. The ADNI study was approved by the Institutional Review Boards of participating institutions, and written informed consent was obtained from all participants or their legally authorized representatives. The present secondary analysis was conducted under the ADNI Data Use Agreement, and no additional local ethics approval was required.

### 2.2. Notation and assumptions

For simplicity, each individual has two potential outcomes: $Y_{i}(\text{1})$ if treated, and $Y_{i}(\text{0})$ if untreated. Under standard causal assumptions for observational data, including consistency, the stable unit treatment value assumption, unconfoundedness, and positivity [13,21], the causal effect of interest is identifiable as Δ=𝔼(Y(1))−E(Y(0)).

Let $Z_{i}$ indicate exposure status and $X_{i}$ denote the vector of observed covariates. The propensity score (PS) is defined as the conditional probability of receiving exposure given covariates, $e\left(X_{i}\right)=P\left(Z_{i}=\text{1}|{\;X}_{i}\right)$. The outcome regression (OR) function is considered as: $\mu_{z}(x)=E(Y|Z=z,X=x)$**,** z∈{0,1}.

To enhance robustness against potential model misspecification, the DR estimator has been proposed in the standard augmented inverse probability weighted form:


$$\begin{array}{c} {\hat{\Delta }}_{\text{DR}}=\frac{\text{1}}{n}\sum_{i=\text{1}}^{n}\left[{\hat{\mu }}_{\text{1}}(X_{i})-{\hat{\mu }}_{\text{0}}(X_{i})+\frac{Z_{i}\left\{Y_{i}-{\hat{\mu }}_{\text{1}}\left(X_{i}\right)\right\}}{\hat{e}\left(X_{i}\right)}-\frac{\left(\text{1}-Z_{\text{i}}\right)\left\{Y_{i}-{\hat{\mu }}_{\text{0}}\left(X_{i}\right)\right\}}{\text{1}-\hat{e}\left(X_{i}\right)}\right]. \end{array}$$

The key property of the DR estimator is that as long as either the PS model or the OR model is correctly specified, the DR estimator remains consistent.

### 2.3. Proposed method

We consider two related domains, a source domain S and a target domain T. Following the transfer causal learning framework, the source and target domains are assumed to share the same covariate space and to have sufficient covariate overlap in applications, while they are allowed to have different feature distributions, treatment assignment mechanisms, and causal effects.

Let the target domain dataset be ${\left\{\left(x_{i,t},z_{i,t},y_{i,t}\right)\right\}}_{i=\text{1}}^{n_{t}}$, where $x_{i,t}\in R^{p}$ denotes the covariates, $z_{i,t}\in \{\text{0},\text{1}\}$ is a binary exposure variable, and $y_{i,t}\in R$ is a continuous outcome variable. Similarly, suppose the source domain dataset is ${\left\{\left(x_{i,s},z_{i,s},y_{i,s}\right)\right\}}_{i=\text{1}}^{n_{s}}$, with $n_{t}<n_{s}$.

Our goal is to estimate the causal effect in the target domain, where the limited sample size makes traditional estimation biased and unstable if relying solely on target data. Because exposure mechanisms differ across domains, direct pooling is invalid, and we therefore adopt a transfer learning approach to borrow information from the source domain while preserving causal validity.

#### 2.3.1 Transfer learning for propensity score model parameters.

Let the PS models in the two domains be defined as:


$$\begin{array}{c} \;P\left(Z_{t}=\text{1}|X_{t}\right)=e_{t}\left(X_{t}^{T}\beta_{t}\right),\;P\left(Z_{s}=\text{1}|X_{s}\right)=e_{s}\left(X_{s}^{T}\beta_{s}\right), \end{array}$$

where $e_{t}(\cdot )$, $e_{s}(\cdot )$ are known link functions, and $\beta_{t}$, $\beta_{s}\in R^{p}$ are domain-specific parameters. In this study, we used the logit link for propensity score modeling in both the source and target domains because the exposure is binary in both domains. This choice was made for simplicity and interpretability, and identical link functions are not required by the h-transferability assumption. Following Wei et al., knowledge transfer does not require the same link function across domains as long as the link functions are known [20]. Meanwhile, the source-target parameter difference is defined as $\delta_{\beta }=\beta_{t}-\beta_{s}.$ The success of transfer learning relies on the sparsity assumption: if ${∥\delta_{\beta }∥}_{\text{0}}\leq h$ for some small constant h, then the exposure assignment mechanisms across domains differ only in a limited subset of covariates. This property is termed **h-transferability**, under which source domain information can be effectively adapted to the target domain.

The estimation proceeds in two steps:

1 **Initial estimation in the source domain**

The initial parameter estimation of $\beta_{s}$ is obtained by penalized maximum likelihood with abundant source data:


$$\begin{array}{c} \hat{\beta }=\text{arg}\underset{\beta }{\text{min}}\left\{\sum_{i=\text{1}}^{n_{s}}\left[-z_{i,s}x_{i,s}^{T}\beta +(\text{ln}\left(\text{1}+{\text{e}}^{x_{i,s}^{T}\beta }\right)\right]+\lambda_{b}{\left\|\beta \right\|}_{\text{1}}\right\} \end{array}$$

where $\lambda_{b}>\text{0}$ is selected via cross-validation.

2 **Bias correction using target domain data**

The sparse inter-domain difference $\delta_{\beta }$ is estimated via ${\text{𝓁}}_{\text{1}}$-regularization:


$$\begin{array}{c} {\hat{\delta }}_{\beta }=\text{arg}\underset{\Delta_{\beta }}{\text{min}}\left\{\sum_{i=\text{1}}^{n_{t}}\left[-z_{i,t}x_{i,t}^{T}\left({\hat{\beta }}_{s}+\Delta_{\beta }\right)+\text{ln}\left(\text{1}+{\text{e}}^{x_{i,t}^{T}\left({\hat{\beta }}_{s}+\Delta_{\beta }\right)}\right)\right]+\lambda_{ps}{\left\|\Delta_{\beta }\right\|}_{\text{1}}\right\} \end{array}$$

where $\lambda_{ps}>\text{0}$ is a tuning parameter selected via cross-validation. Wei et al. demonstrated the theoretical validity of the Lasso-based transfer estimator under appropriate regularity conditions.

Then, the final PS parameter estimate is:


$$\begin{array}{c} {\hat{\beta }}_{t}={\hat{\beta }}_{s}+{\hat{\delta }}_{\beta } \end{array}$$

**Algorithm 1 Double Shrinkage Transfer Causal Learning Estimation**

**1. Input** target data ${𝔇}_{t}$, source data ${𝔇}_{s}$

**2. Initial estimation in the source domain**

        ${\hat{\theta }}_{s}=\text{argmin }L\left(\theta_{s};{𝔇}_{s}\right)+\lambda_{s}{\left\|\theta_{s}\right\|}_{\text{1}}$

**3. Bias correction using target domain data**

       ${\hat{\delta }}_{\theta }=\text{argmin }L\left\{\left({\hat{\theta }}_{s}+\delta_{\theta }\right);{𝔇}_{t}\right\}+\lambda_{\theta }{\left\|\delta_{\theta }\right\|}_{\text{1}}$

**4. Final estimation in the target domain**

          ${\hat{\theta }}_{t}={\hat{\theta }}_{s}+{\hat{\delta }}_{\theta }$

**5. Output** ${\hat{\theta }}_{t}$

#### 2.3.2. Transfer learning for outcome model parameters.

Analogously, the OR models are specified separately for each group


$$\begin{array}{c} \mu_{z,t}(x_{t})=E\left[Y_{t}|Z_{t}=z,\;X_{t}=x_{t}\right]=m_{z,t}\left(x_{t};\alpha_{z,t}\right),\; \end{array}$$

$$\begin{array}{c} \mu_{z,s}(x_{s})=E\left[Y_{s}|Z_{s}=z,\;X_{s}=x_{s}\right]=m_{z,s}\left(x_{s};\alpha_{z,s}\right), \end{array}$$

where $m_{z,t}\left(\cdot \right)$ and $m_{z,s}\left(\cdot \right)$ are functions with known forms, whereas $\alpha_{z,t}$ and $\alpha_{z,s}$ represent the unknown parameters. For simplicity, we parameterize the conditional expectations in both domains via linear regression models as follows:


$$\begin{array}{c} \mu_{z,t}(x_{t})=E\left[Y_{t}|Z_{t}=z,\;X_{t}=x_{t}\right]=x_{t}^{T}\alpha_{z,t}\; \end{array}$$

$$\begin{array}{c} \;\mu_{z,s}(x_{s})=E\left[Y_{s}|Z_{s}=z,\;X_{s}=x_{s}\right]=x_{s}^{T}\alpha_{z,s} \end{array}$$

As long as the forms of $m_{z,t}\left(\cdot \right)$ and $m_{z,s}\left(\cdot \right)$ are known, the learned components remain independent of the assumption that the “means” follow linear regression conditions.

For OR models, the source-target parameter difference is given by:


$$\begin{array}{c} \delta_{z,\alpha }=\alpha_{z,t}\;-\alpha_{z,s} \end{array}$$

The joint transportability of the model parameters similarly requires sparsity of $\delta_{z,\alpha }$. For z∈{0,1}, denote $n_{z,s}=\#\{i:\;z_{i,s}=z\}\;$and $n_{z,t}=\#\{i:\;z_{i,t}=z\}$, where #{·} represents the cardinality of a set. This allows us to apply the aforementioned regularized transfer learning techniques, leveraging source domain data to estimate the OR parameters in the target domain:


$$\begin{array}{c} {\hat{\alpha }}_{z,s}=\text{arg}\underset{\alpha }{\text{min}}\left[\frac{\text{1}}{n_{z,s}}\sum_{z_{i,s}=z}{\left(y_{i,s}-x_{i,s}^{T}\alpha \right)}^{\text{2}}+\lambda_{s}\|\;\alpha \|\;\text{1}\right] \end{array}$$

$$\begin{array}{c} {\hat{\delta }}_{z,\alpha }=\text{arg}\underset{\Delta_{z,\alpha }}{\text{min}}\left[\frac{\text{1}}{n_{z,t}}\sum_{z_{i,t}=z}{\left(y_{i,t}-x_{i,t}^{T}\left({\hat{\alpha }}_{z,s}+\Delta_{z,\alpha }\right)\right)}^{\text{2}}+\lambda_{or}{\left\|\Delta_{z,\alpha }\right\|}_{\text{1}}\right] \end{array}$$

$$\begin{array}{c} {\hat{\alpha }}_{z,t}={\hat{\alpha }}_{z,s}+{\hat{\delta }}_{z,\alpha } \end{array}$$

where ${\lambda_{s},\;\lambda }_{or}>\text{0}$ are selected via cross-validation.

#### 2.3.3 Estimation of causal effect.

With transfer-learned parameters in PS and OR models, we construct the DSTCL estimator as follows, with detailed steps in Algorithm 2:


$$\begin{array}{c} {\hat{\Delta }}_{\text{DSTCL}}=\frac{\text{1}}{n_{t}}\sum_{i=\text{1}}^{n_{t}}\left[{\hat{\mu }}_{\text{1},\text{DSTCL}}(x_{i})-{\hat{\mu }}_{\text{0},\text{DSTCL}}(x_{i})+\frac{Z_{i}\left\{Y_{i}-{\hat{\mu }}_{\text{1},\text{DSTCL}}\left(x_{i}\right)\right\}}{{\hat{e}}_{\text{DSTCL}}\left(x_{i}\right)}-\frac{\left(\text{1}-Z_{\text{i}}\right)\left\{Y_{i}-{\hat{\mu }}_{\text{0},\text{DSTCL}}\left(x_{i}\right)\right\}}{\text{1}-{\hat{e}}_{\text{DSTCL}}\left(x_{i}\right)}\right] \end{array}$$

**Algorithm 2 Proposed DSTCL Estimator of causal effect**

**1. Input** target data ${\left\{\left(x_{i,t},z_{i,t},y_{i,t}\right)\right\}}_{i=\text{1}}^{n_{t}}$, source data${\left\{\left(x_{i,s},z_{i,s},y_{i,s}\right)\right\}}_{i=\text{1}}^{n_{s}}$

**2. Transfer learning for propensity score**

       $\begin{array}{c} {\hat{\beta }}_{s}=\text{arg}\underset{\beta }{\text{min}}\left\{\sum_{i=\text{1}}^{n_{s}}\left[-z_{i,s}x_{i,s}^{T}\beta +\text{ln}\left(\text{1}+{\text{e}}^{x_{i,s}^{T}\beta }\right)\right]+\lambda_{b}{\left\|\beta \right\|}_{\text{1}}\right\} \end{array}$

   ${\hat{\delta }}_{\beta }=\text{arg}\underset{\Delta_{\beta }}{\text{min}}\left\{\sum_{i=\text{1}}^{n_{t}}\left[-z_{i,t}x_{i,t}^{T}\left({\hat{\beta }}_{s}+\Delta_{\beta }\right)+\text{ln}\left(\text{1}+{\text{e}}^{x_{i,t}^{T}\left({\hat{\beta }}_{s}+\Delta_{\beta }\right)}\right)\right]+\lambda_{ps}{\left\|\Delta_{\beta }\right\|}_{\text{1}}\right\}$

             ${\hat{\beta }}_{t}={\hat{\beta }}_{s}+{\hat{\delta }}_{\beta }$

3. **Transfer learning for outcome regression**

       ${\hat{\alpha }}_{z,s}=\text{arg}\underset{\alpha }{\text{min}}\left[\frac{\text{1}}{n_{z,s}}\sum_{z_{i,s}=z}{\left(y_{i,s}-x_{i,s}^{T}\alpha \right)}^{\text{2}}+\lambda_{s}\|\;\alpha \|\;\text{1}\right]$

    ${\hat{\delta }}_{z,\alpha }=\text{arg}\underset{\Delta_{z,\alpha }}{\text{min}}\left[\frac{\text{1}}{n_{z,t}}\sum_{z_{i,t}=z}{\left(y_{i,t}-x_{i,t}^{T}\left({\hat{\alpha }}_{z,s}+\Delta_{z,\alpha }\right)\right)}^{\text{2}}+\lambda_{or}{\left\|\Delta_{z,\alpha }\right\|}_{\text{1}}\right]$

             ${\hat{\alpha }}_{z,t}={\hat{\alpha }}_{z,s}+{\hat{\delta }}_{z,\alpha }$

**4. Construct DR estimator in target domain**

           ${\hat{\Delta }}_{\text{DSTCL}}=\frac{\text{1}}{n_{t}}\sum_{i=\text{1}}^{n_{t}}\left[{\hat{\mu }}_{\text{1},\text{DSTCL}}(x_{i})-{\hat{\mu }}_{\text{0},\text{DSTCL}}(x_{i})+\frac{Z_{i}\left\{Y_{i}-{\hat{\mu }}_{\text{1},\text{DSTCL}}\left(x_{i}\right)\right\}}{{\hat{e}}_{\text{DSTCL}}\left(x_{i}\right)}-\frac{\left(\text{1}-Z_{\text{i}}\right)\left\{Y_{i}-{\hat{\mu }}_{\text{0},\text{DSTCL}}\left(x_{i}\right)\right\}}{\text{1}-{\hat{e}}_{\text{DSTCL}}\left(x_{i}\right)}\right]$

5.**Output** ${\hat{\Delta }}_{\text{DSTCL}}$

The proposed DSTCL framework assumes that the source and target domains share the same covariate space and relies on standard causal assumptions, including consistency, unconfoundedness, and positivity, as well as transfer-learning assumptions including sufficient source-target covariate overlap and sparsity of source-target parameter differences. These assumptions are consistent with existing transfer learning theory, where theoretical guarantees generally require source-target similarity, sparsity, and regularity conditions [26,27]. In our setting, these assumptions allow information from the source domain to improve parameter estimation in the target domain while preserving the target domain causal estimand.

All *l*_1_-regularized nuisance models were fitted using glmnet with alpha = 1, and tuning parameters were selected by cross-validation. We used five-fold cross-validation whenever feasible, with binomial deviance for propensity score models and mean squared error for outcome regression models. In DSTCL, tuning was performed separately for each nuisance component, and the source domain initial estimation step and target domain correction step used separate cross-validation procedures. The primary analysis used lambda.1se, with lambda.min considered in sensitivity analyses.

## 3. Simulation

### 3.1. Simulation design

We evaluated the proposed method using simulated data with a target sample size $n_{t}=\text{50}$ and a source sample size $n_{s}=\text{1000}$, and p=200 covariates in both domains. For each individual i in both the source and target domains, the covariates $x_{i}$ are generated from a multivariate normal distribution:


$$\begin{array}{c} x_{i}\sim N\left(0_{p},\;\Sigma \right),\;\Sigma =\left(\Sigma_{jk}\right),\;\Sigma_{jk}={\text{0}.\text{2}}^{|j-k|+\text{1}},\;i=\text{1},\;\text{2},\;\cdots ,\;n. \end{array}$$

The design matrix is constructed as $X={(x_{\text{1}},\;x_{\text{2}},\cdots ,x_{\text{n}})}^{T}$.

A binary variable is generated based on these covariates, with the true PS models specified as follows:


$$\begin{array}{c} \text{logit}\left\{\text{P}\left(Z_{t}=\text{1}|X_{t}\right)\right\}=\beta_{\text{0},t}+X_{t}^{T}\beta_{\text{1},t},\;\text{logit}\left\{\text{P}\left(Z_{s}=\text{1}|X_{s}\right)\right\}=\beta_{\text{0},s}+X_{s}^{T}\beta_{\text{1},s}\text{.} \end{array}$$

The parameters are set as $\beta_{\text{1},t}={\left(\underset{\text{5}}{\underbrace{-\text{0}.\text{3},\cdots ,-\text{0}.\text{3}}},\underset{\text{45}}{\underbrace{\text{0}.\text{6},\cdots ,\text{0}.\text{6}}},\underset{\text{150}}{\underbrace{\text{0},\cdots ,\text{0}}}\right)}^{T}$ with $\beta_{\text{0},t}=-\text{0}.\text{2}$ and $\beta_{\text{1},s}={\left(\underset{\text{5}}{\underbrace{\text{0}.\text{3},\cdots ,\text{0}.\text{3}}},\underset{\text{45}}{\underbrace{\text{0}.\text{6},\cdots ,\text{0}.\text{6}}},\underset{\text{150}}{\underbrace{\text{0},\cdots ,\text{0}}}\right)}^{T}$ with $\beta_{\text{0},s}=\text{0}.\text{2}$. The true coefficient differences in the PS models between sourceand target domains are $\delta_{\beta }=(-\text{0}.\text{4},\underset{\text{5}}{\underbrace{-\text{0}.\text{6},\cdots ,-\text{0}.\text{6}}},\underset{\text{195}}{\underbrace{\text{0},\cdots ,\text{0}}}),$ implying the transportability parameter h=6.

The outcome variable Y is defined as:


$$\begin{array}{c} Y_{z,t}=\alpha_{\text{0},z,t}+X_{t}^{T}\alpha_{\text{1},t}+\varepsilon_{z,t},\;Y_{z,s}=\alpha_{\text{0},z,s}+X_{s}^{T}\alpha_{\text{1},s}+\varepsilon_{z,s},\text{ z}\in \{\text{0},\text{1}\}\text{.} \end{array}$$

where the residuals in both groups across domains follow a normal distribution $N\left(\text{0},{\text{0}.\text{5}}^{\text{2}}\right)$. Additionally, for the target domain, the parameters are set as $\alpha_{\text{1},t}={\left(\underset{\text{5}}{\underbrace{-\text{0}.\text{3},-\text{0}.\text{3},-\text{0}.\text{3},\text{0}.\text{3},\text{0}.\text{3}}},\underset{\text{25}}{\underbrace{\text{0}.\text{8},\cdots ,\text{0}.\text{8}}},\underset{\text{20}}{\underbrace{-\text{0}.\text{8},\cdots ,-\text{0}.\text{8}}},\underset{\text{150}}{\underbrace{\text{0},\cdots ,\text{0}}}\right)}^{T},$ with intercept $\alpha_{\text{0},\text{1},t}=-\text{1}$ and $\alpha_{\text{0},\text{0},t}=\text{1},$ yielding a true causal effect of $\Delta_{t}=-\text{2}$. For the source domain, the parameters are set as $\alpha_{\text{1},s}={\left(\underset{\text{5}}{\underbrace{\text{0}.\text{3},\text{0}.\text{3},\text{0}.\text{3},-\text{0}.\text{3},-\text{0}.\text{3}}},\underset{\text{25}}{\underbrace{\text{0}.\text{8},\cdots ,\text{0}.\text{8}}},\underset{\text{20}}{\underbrace{-\text{0}.\text{8},\cdots ,-\text{0}.\text{8}}},\underset{\text{150}}{\underbrace{\text{0},\cdots ,\text{0}}}\right)}^{T},$ with intercept $\alpha_{\text{0},\text{1},s}=\text{1}$ and$\alpha_{\text{0},\text{0},s}=-\text{1},$ then the true causal effect is $\Delta_{\text{s}}=\text{2}$. Then the true coefficient differences between two domains are$\delta_{z,\alpha }=(\text{2}-\text{4z},\underset{\text{5}}{\underbrace{-\text{0}.\text{6},-\text{0}.\text{6},-\text{0}.\text{6},\text{0}.\text{6},\text{0}.\text{6}}},\underset{\text{195}}{\underbrace{\text{0},\cdots ,\text{0}}})$, implying the transportability parameter for the outcome model is also 6.

To assess robustness under model misspecification, we considered two regimes: a PS-correct setting, where the exposure model is correctly specified but the outcome model includes a nonlinear sine term, and an OR-correct setting, where the outcome model is correct but the exposure model is misspecified by omitting covariates and adding nonlinearity. Simulations were repeated 200 times, with performance metrics evaluated based on these simulated datasets. We also recorded computational runtime and convergence status for each simulation replication.

We compared the following 8 methods:

1. **Crude difference (Crude_diff)**: The unadjusted mean difference in outcomes between the treated and control groups in the target domain.
2. **Known parameters (Known_param)**: A doubly robust estimator in which the true PS and OR model parameters are plugged in, serving as a benchmark.
3. **Generalized linear model (GLM)**: The DR estimator with nuisance parameters estimated in the target domain using traditional logistic regression for PS and linear regression for OR model.
4. **Least absolute shrinkage and selection operator (Lasso)**: The DR estimator with parameters estimated in the target domain via Lasso regularization.
5. **Double Machine Learning (DML)**: A cross-fitted causal learning framework that estimates the PS and OR model using machine learning methods.
6. **Targeted Maximum Likelihood Estimation (TMLE)**: A doubly robust procedure that updates an initial OR model using information from PS to target the parameter of interest; both the initial PS and OR model are fitted with Lasso regularization.
7. $l_{\text{1}}$**-Transfer causal learning estimator (**$l_{1}$**-TCL)**: A transfer learning approach in which models are first initialized using MLE in source domain, then bias-corrected in target domain via Lasso, and finally substituted into the DR estimator.
8. **Double Shrinkage Causal Transfer Learning (DSTCL)**: Our proposed transfer causal learning estimator.

Furthermore, the evaluation of estimator performance was based on the following criteria:

1. **Bias:** The Bias of overall causal effect was calculated across 200 replications as: $Bias=\frac{\text{1}}{\text{200}}\sum_{\text{i}=\text{1}}^{\text{200}}\left({\hat{\theta }}_{i}-\theta \right)$, where θ denotes the true value of causal effect, and ${\hat{\theta }}_{i}$ represents the estimated value obtained in the i-th replication.
2. **Standard deviation (SD):** The standard deviation of the estimator across 200 replications was computed as:$SD=\sqrt{\frac{\text{1}}{\text{200}-\text{1}}\sum_{i=\text{1}}^{\text{200}}{\left({\hat{\theta }}_{i}-\overset{―}{\theta }\right)}^{\text{2}}},$ where $\overset{―}{\theta }=\frac{\text{1}}{\text{200}}\sum_{\text{i}=\text{1}}^{\text{200}}{\hat{\theta }}_{i}$ denotes the average estimate across the 200 replications.
3. **Mean squared error (MSE):** The mean squared error of the estimator was assessed over 200 replications as:

$$\begin{array}{c} MSE=\frac{\text{1}}{\text{200}}\sum_{\text{i}=\text{1}}^{\text{200}}{\left({\hat{\theta }}_{i}-\overset{―}{\theta }\right)}^{\text{2}} \end{array}$$

4 **Coverage probability of the 95% percentile confidence interval (Coverage)**: For each of the 200 replications, a95% percentile bootstrap confidence interval was constructed based on 500 bootstrap resamples, defined as${CI}_{i}=\left[{\hat{\theta }}_{\text{i},\text{0}.\text{025}},\;{\hat{\theta }}_{\text{i},\text{0}.\text{975}}\right]$. Coverage was then evaluated by checking whether the true parameter θ fell within theconfidence interval, with ${\text{Cover}}_{i}=\left\{ \begin{array}{c} \text{1},\;if\;\theta \in {\text{CI}}_{i}\;\\ \text{0},\text{ otherwise } \end{array}\right.\;$. Finally, the overall coverage probability was obtained as:

$$\begin{array}{c} \text{95}\%\text{ Cov}=\frac{\text{1}}{\text{200}}\sum_{i=\text{1}}^{\text{200}}{\text{Cover}}_{i} \end{array}$$

### 3.2. Simulation scenarios

**Scenario 1. Effect of source domain sample size**: In this scenario, we investigated how gradual increases in source domain sample size affected the performance of the DSTCL estimator. Target domain sample size was fixed at $n_{t}=\text{50}$, while source domain sample size varied as $n_{s}\in \{\text{500},\text{1000},\text{2000}\}$.

**Scenario 2. Effect of inter-domain similarity**: To evaluate the effect of inter-domain similarity, we further varied h∈{6,11,16}, which quantifies the number of nonzero elements in parameter difference vectors. Smaller h corresponds to stronger similarity between source and target domains, while larger h reflects weaker transferability. All other settings remained unchanged.

Scenario 3. Effect of the number of covariates: In this setting, we examined the sensitivity of different estimators to increasing dimensionality by systematically varying the covariate size. Specifically, we considered p ∈ {50,100,200} while holding all other simulation parameters fixed.

We also conducted several sensitivity analyses under representative settings to evaluate robustness to the penalty-selection rule, extreme propensity scores, propensity-score trimming, and mild covariate shift between the source and target domains. Specifically, we compared lambda.1se with lambda.min, increased the propensity-score strength parameter to generate probabilities closer to 0 or 1, compared no trimming with propensity score truncation to [0.01, 0.99] and [0.05, 0.95], and introduced mild target domain covariate mean shift. Details are provided in the Supporting Information.

### 3.3. Simulation results

In the primary simulation setting with $n_{t}=50,\;n_{s}=1000,\;p=200,\;h=6$, the proposed DSTCL estimator showed performance close to the oracle benchmark and clearly superior to other methods (Table 1). When both the PS and OR models were correctly specified, DSTCL exhibited negligible Bias and the smallest SD among all data-driven estimators, leading to the lowest MSE and coverage close to 95%. In contrast, Lasso, DML1 (Lasso-randomforest), DML2 (SVM-randomforest), and TMLE all displayed either larger absolute Bias, inflated SD, or both, which translated into higher MSEs, while $l_{1}$-TCL failed to yield an estimate. Similar patterns were observed when only the PS or OR model was correctly specified. DSTCL consistently achieved small Bias and relatively tight variability, with MSE values comparable to or smaller than those of the oracle estimator and coverage remaining near 0.95. In this primary simulation setting, DSTCL required approximately 1.71 seconds per main fit and 13.96 seconds per individual bootstrap refit on average; thus, 500 bootstrap resamples required approximately 6,980 seconds. No DSTCL estimation failures were observed across the 200 simulation replications.

**Table 1:** The performance across different methods in primary simulation setting.

	Estimator	Bias (SD)	MSE	Coverage
**Both the PS and OR models are correct**	**Crude_diff**	0.42 (0.87)	0.93	0.92
	**Known_param**	0.00 (0.33)	0.11	0.94
	**Glm**	–	–	–
	**Lasso**	0.45 (0.82)	0.87	0.78
	**DML1**	0.28 (0.79)	0.69	0.92
	**DML2**	0.25 (0.83)	0.76	0.92
	**TMLE**	0.34 (0.84)	0.82	0.99
	$l_{1}$ **-TCL**	–	–	–
	**DSTCL**	**0.06 (0.26)**	**0.07**	**0.94**
**Only the PS model is correct**	**Crude_diff**	1.22 (0.45)	1.69	0.26
	**Known_param**	0.19 (1.60)	2.58	0.90
	**Glm**	–	–	–
	**Lasso**	1.21 (0.44)	1.65	0.06
	**DML1**	1.20 (0.54)	1.72	0.28
	**DML2**	1.19 (0.52)	1.68	0.27
	**TMLE**	0.89 (0.51)	1.05	0.73
	$l_{1}$ **-TCL**	–	–	–
	**DSTCL**	**0.12 (0.24)**	**0.07**	**0.92**
**Only the OR model is correct**	**Crude_diff**	0.36 (0.86)	0.87	0.94
	**Known_param**	0.02 (0.32)	0.10	0.90
	**Glm**	–	–	–
	**Lasso**	0.41 (0.79)	0.79	0.80
	**DML1**	0.53 (0.84)	0.97	0.87
	**DML2**	0.52 (0.83)	0.96	0.88
	**TMLE**	0.52 (0.92)	1.12	0.96
	$l_{1}$ **-TCL**	–	–	–
	**DSTCL**	**0.07 (0.25)**	**0.07**	**0.96**

**Fig 1:**
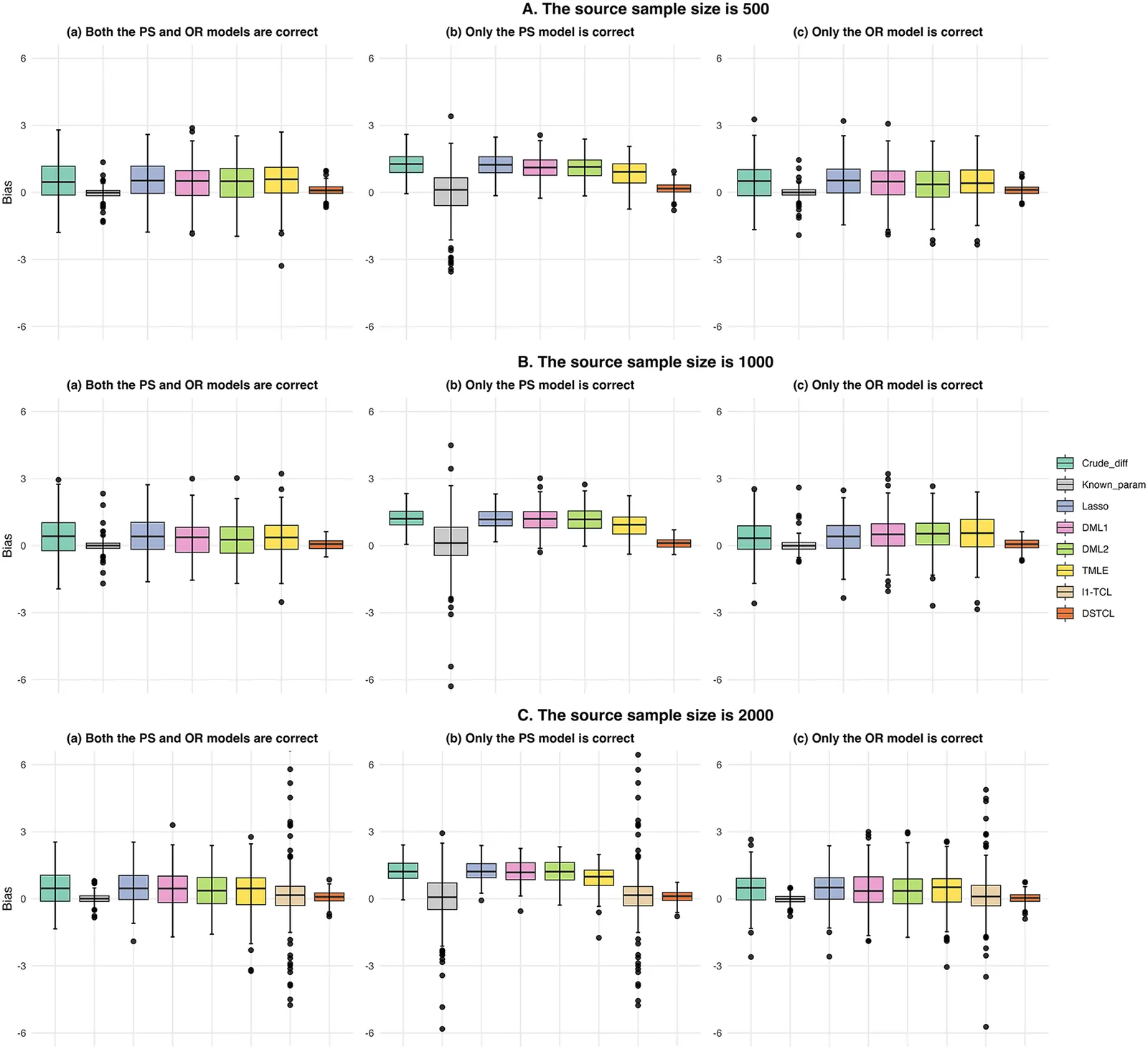
Boxplots of Bias and SD for varying the sample size in source domain (Scenario 1).

**Fig 2:**
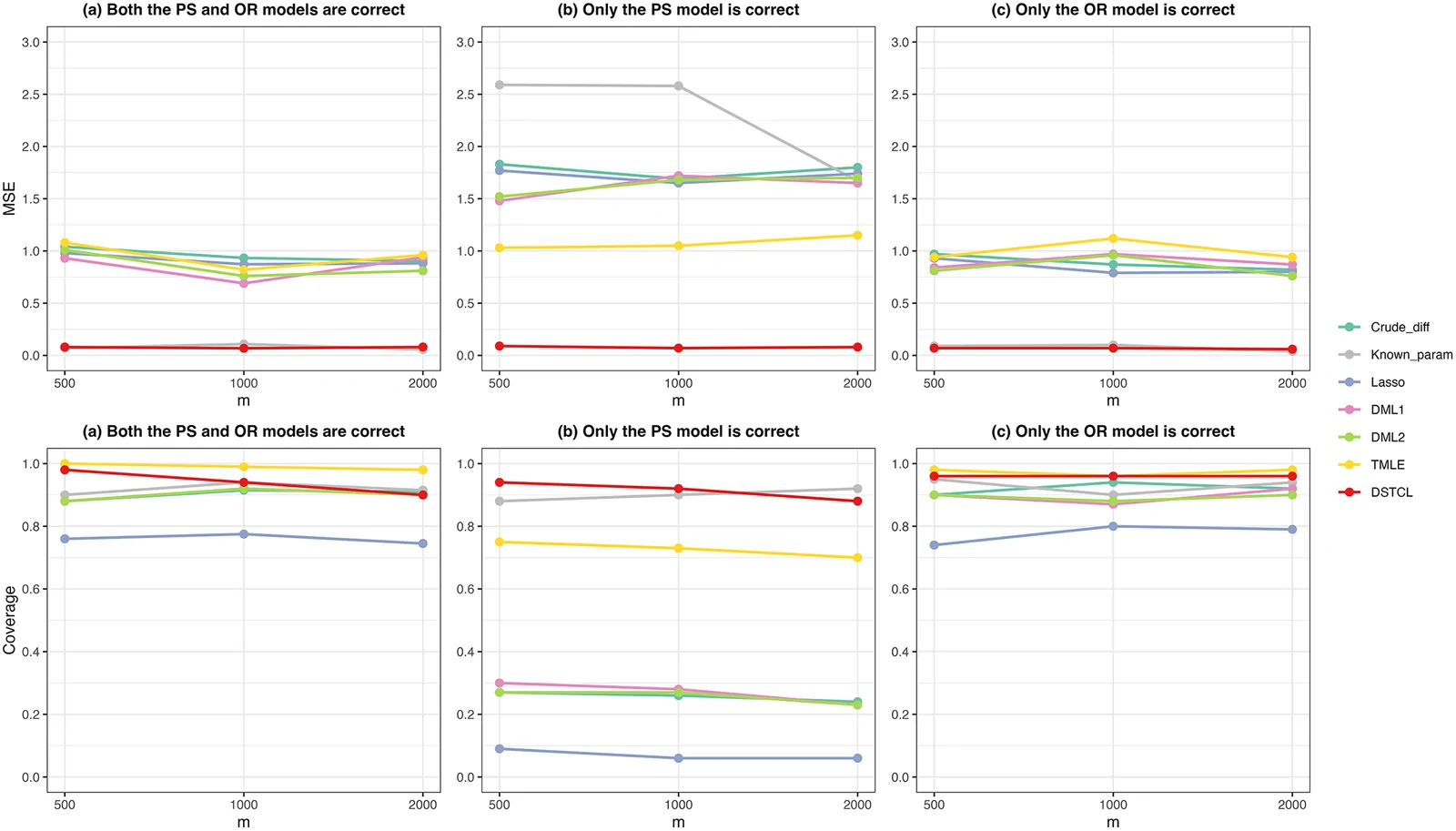
Line plots of MSE and Coverage for varying the sample size in source domain (Scenario 1). *l*_1_-TCL was omitted from the line plots because complete simulation and bootstrap results were not available across all settings. Available *l*_1_-TCL results are reported in the corresponding Supporting Information tables.

As $n_{s}$ increased from 500 to 1000 and 2000, the proposed DSTCL Bias remained very close to zero and SD changed only modestly, leading to uniformly low MSE (Figs 1 and 2 and Table A in S1 Appendix). Across these settings, its bias ranged from 0.04 to 0.17, its MSE ranged from 0.06 to 0.09, and its coverage ranged from 0.88 to 0.98. In contrast, the crude difference, Lasso, DML1, DML2, and TMLE estimators exhibited persistently larger absolute Bias and/or inflated variability, yielding substantially higher MSEs even when either the PS or OR model was correctly specified. Moreover, $l_{1}$-TCL failed to produce valid estimates in a non-negligible fraction of replicates when the source-domain sample size was 500 or 1000, but converged reliably when $n_{s}=2000$, indicating that its numerical stability requires a sufficiently large source dataset. These results indicate that DSTCL can reliably exploit auxiliary information from source domain even when the source sample size is moderate, and that it does not rely on a very large source cohort to achieve clear improvements.

**Fig 3:**
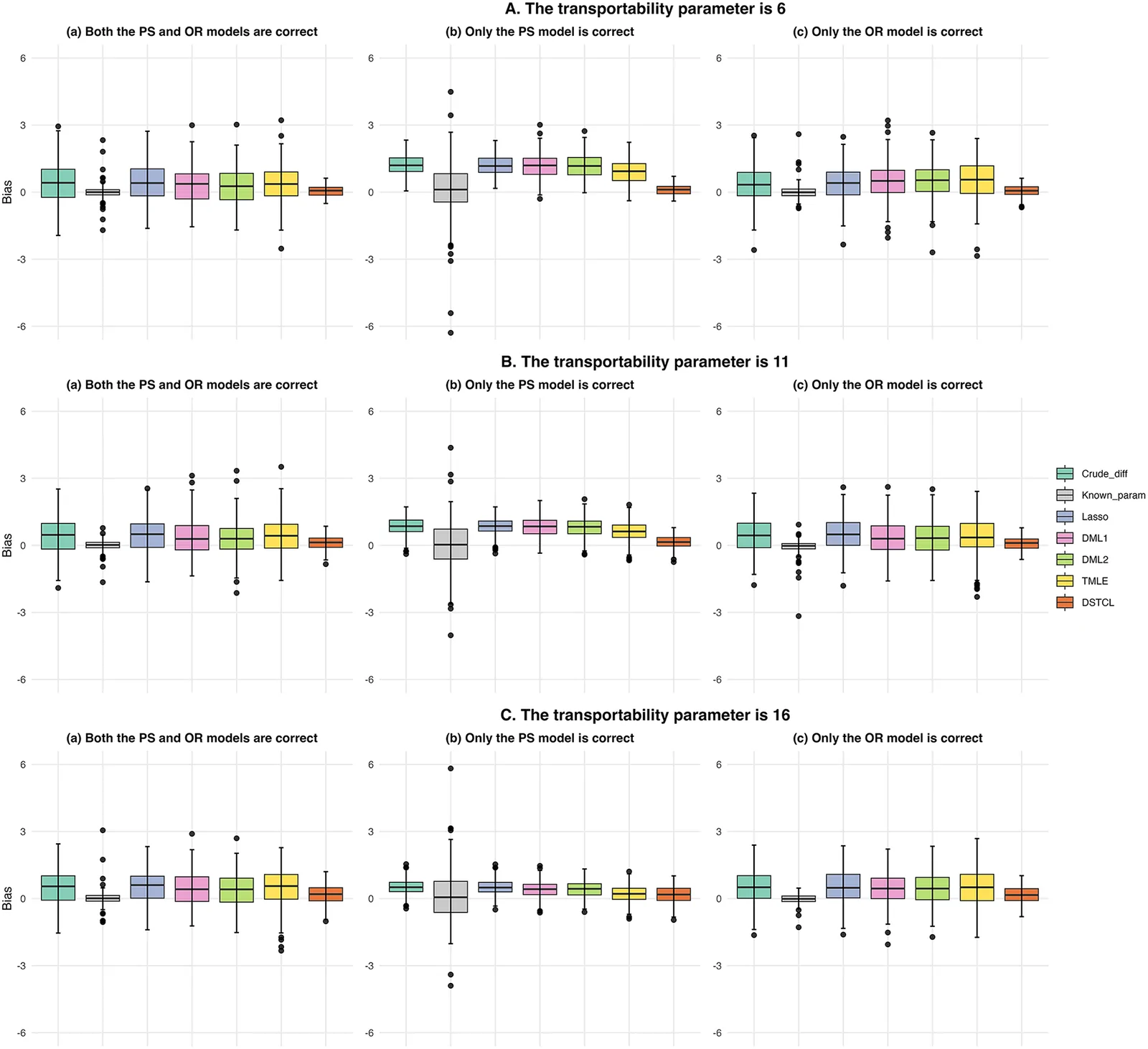
Boxplots of Bias and SD for varying the h-transferability (Scenario 2).

**Fig 4:**
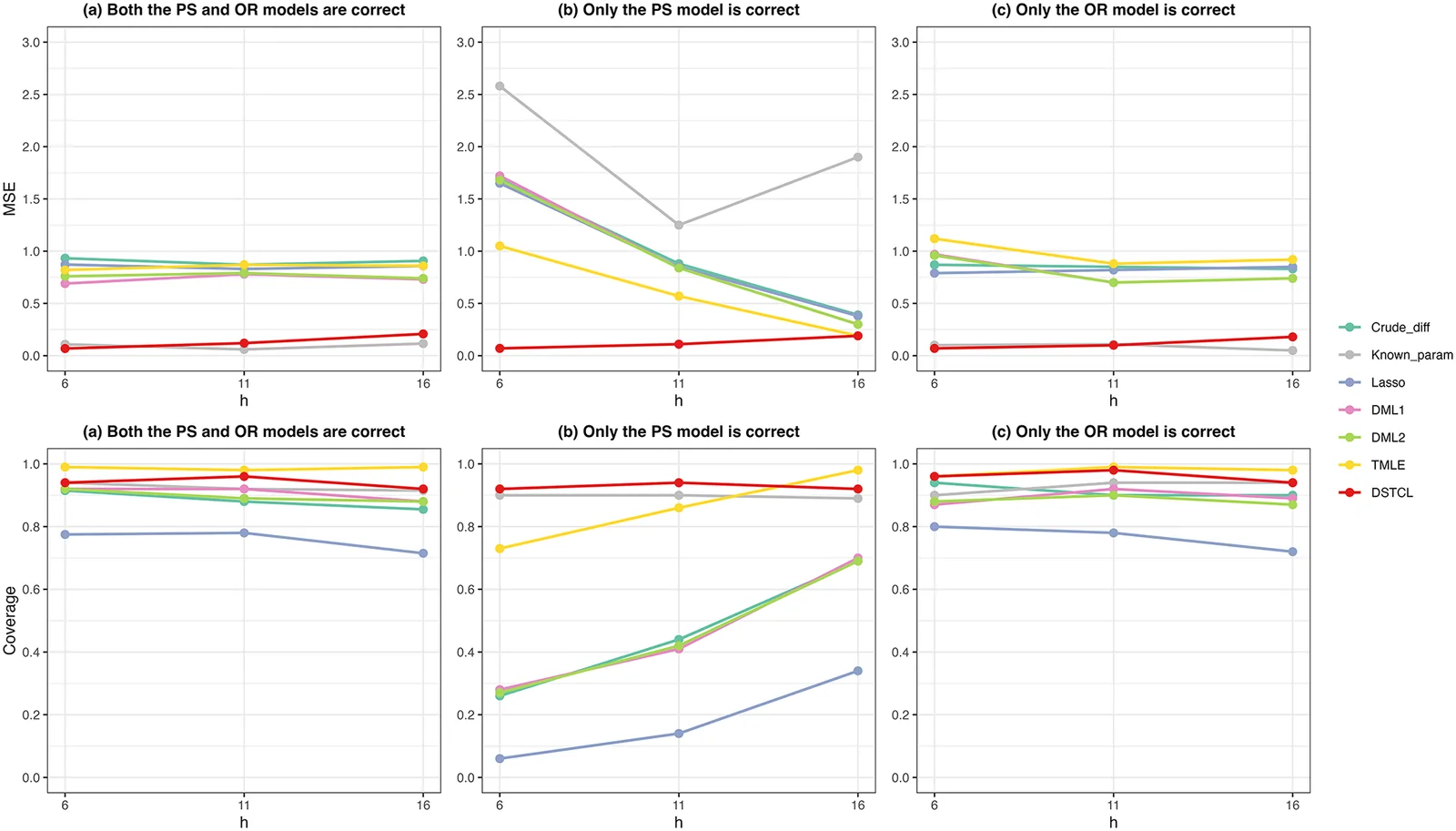
Line plots of MSE and Coverage for varying the h-transferability (Scenario 2). *l*_1_-TCL was omitted from the line plots because complete simulation and bootstrap results were not available across all settings. Available *l*_1_-TCL results are reported in the corresponding Supporting Information tables.

In Scenario 2, we examined the impact of inter-domain similarity by varying h (Figs 3 and 4 and Table B in S1 Appendix). When h=6, corresponding to relatively strong similarity between domains, DSTCL again achieved near-oracle Bias and the lowest MSE among all estimators. As h increased to 11 and 16, the absolute Bias and variability of DSTCL increased only modestly and remained much smaller than those of target-only estimators. Across the three model-specification scenarios, DSTCL had bias ranging from 0.06 to 0.19, MSE ranging from 0.07 to 0.21, and coverage ranging from 0.92 to 0.98. In contrast, complete $l_{1}$-TCL results were not available across these settings. These findings suggest that DSTCL provides robust inference when cross-domain parameter differences are relatively large.

**Fig 5:**
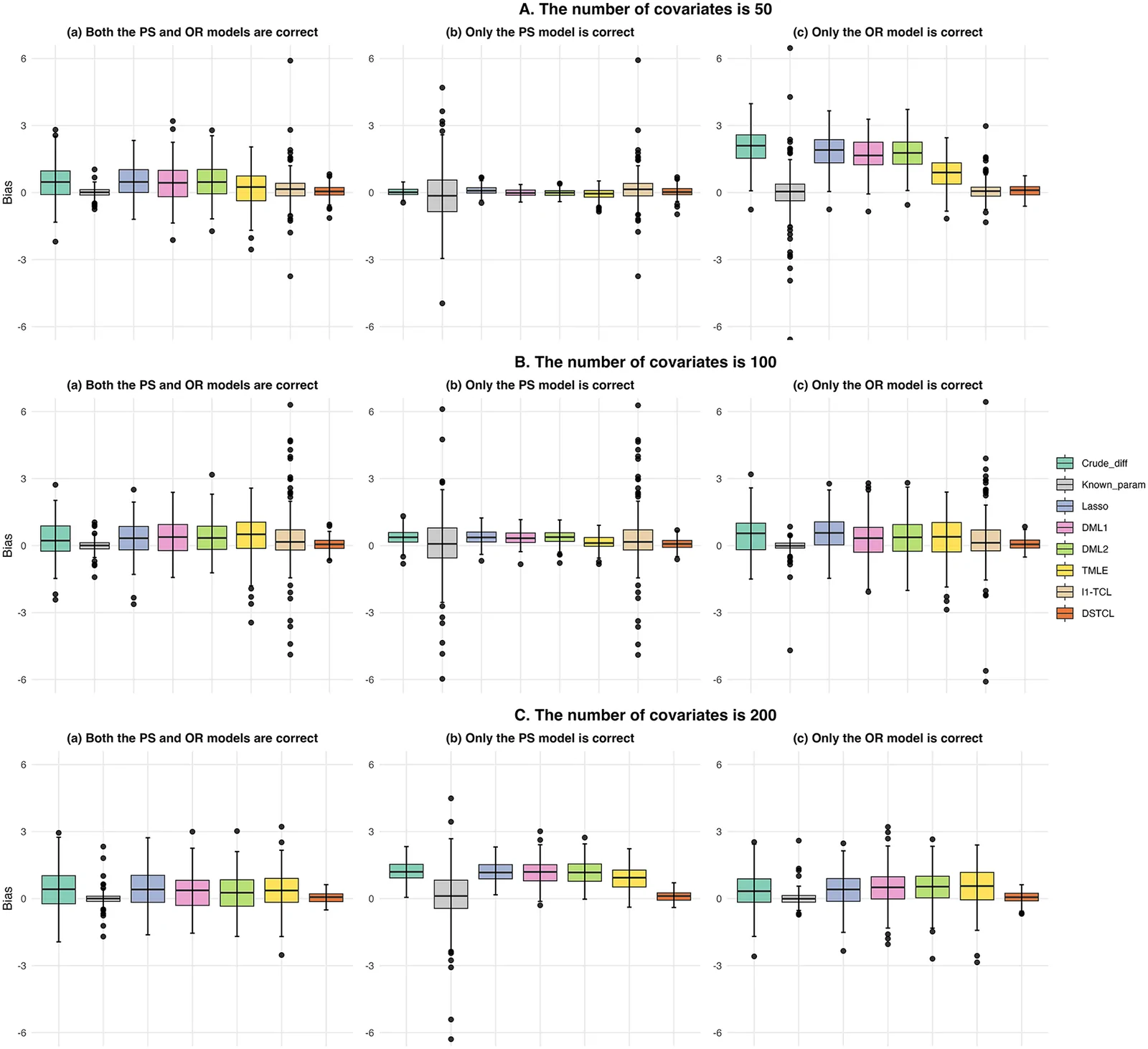
Boxplots of Bias and SD for varying the number of covariates (Scenario 3).

**Fig 6:**
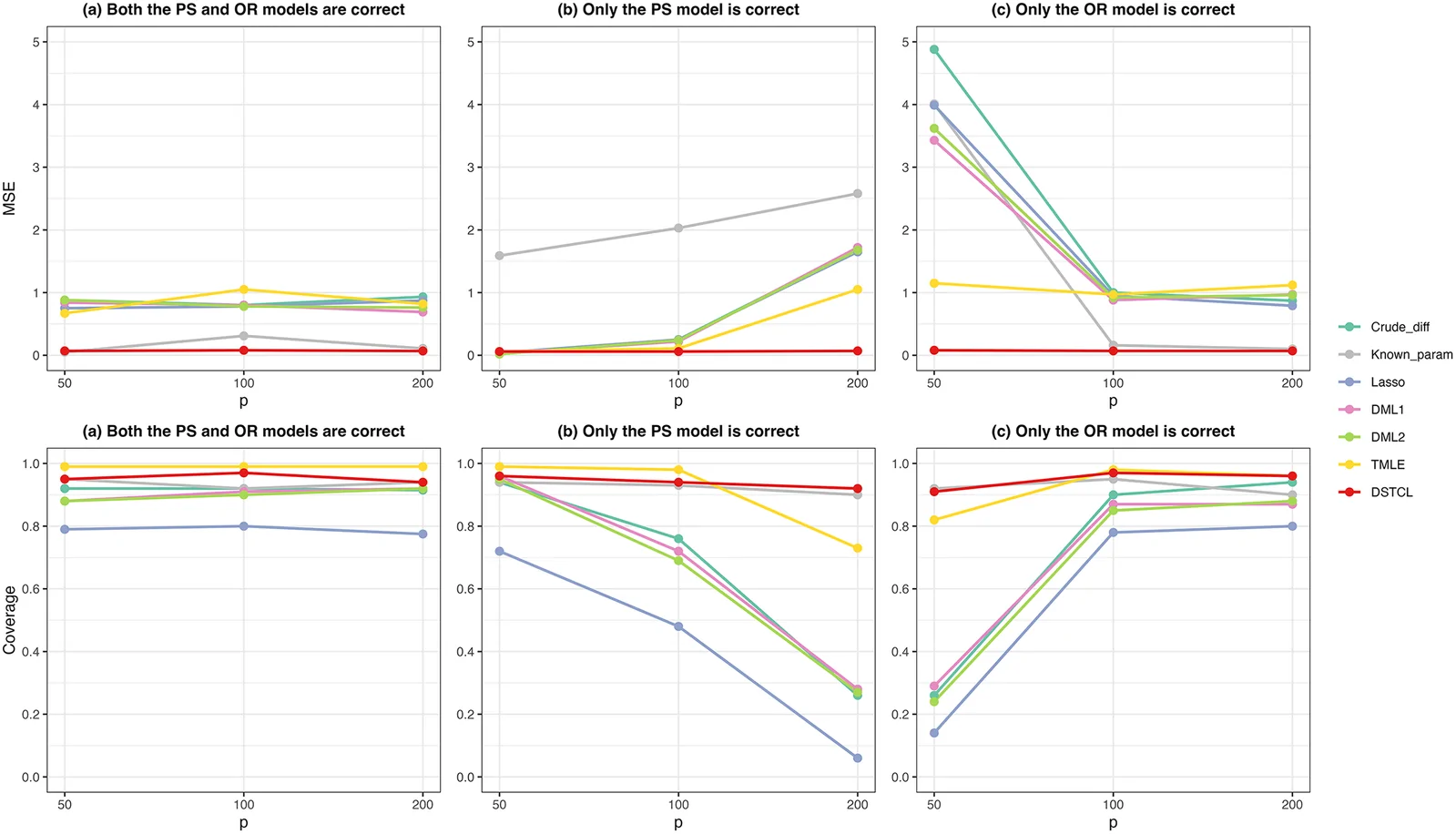
Line plots of MSE and Coverage for varying the number of covariates (Scenario 3). *l*_1_-TCL was omitted from the line plots because complete simulation and bootstrap results were not available across all settings. Available *l*_1_-TCL results are reported in the corresponding Supporting Information tables.

When the covariate dimension was increased, the performance of the DSTCL estimator was essentially unchanged (Figs 5 and 6 and Table C in S1 Appendix). Across the three model-specification scenarios, its bias ranged from 0.04 to 0.12, its MSE ranged from 0.06 to 0.08, and its coverage ranged from 0.91 to 0.97. Although some competing estimators achieved comparable or lower MSE in the PS-correct setting when *p* = 50, their performance varied more substantially across covariate dimensions and model-specification scenarios. In most settings, the competing data-driven estimators exhibited greater variability or larger MSE than DSTCL. In contrast, DSTCL consistently maintained low MSE and coverage close to 95%.

Taken together, these simulations suggest that the proposed DSTCL estimator performs well when the target population has a limited sample size. Under different data generating mechanisms, DSTCL achieved small Bias and low MSE while maintaining approximately 95% coverage.

Additional sensitivity analyses showed that the main conclusions were not highly sensitive to the penalty-selection rule, propensity-score trimming, extreme propensity-score settings, or mild covariate shift. Results obtained using lambda.min were broadly comparable to those from the primary lambda.1se analysis, although some deterioration was observed in the PS-correct setting. Under the most extreme propensity-score setting, DSTCL showed some expected performance degradation but remained relatively stable, with bias ranging from 0.11 to 0.18, MSE ranging from 0.11 to 0.17, and coverage ranging from 0.92 to 0.97. Propensity-score trimming did not materially alter the bias or MSE, although coverage changed modestly in some settings. Under mild covariate shift, bias ranged from 0.05 to 0.15, MSE ranged from 0.08 to 0.10, and coverage ranged from 0.92 to 0.96. Computational diagnostics showed no DSTCL estimation failures across the 200 replications at any evaluated covariate dimension. The mean main-fit time ranged from 0.86 to 2.01 seconds, while the mean time per individual bootstrap refit ranged from 1.03 to 16.04 seconds and increased with the covariate dimension. Detailed sensitivity and computational results are reported in the Supporting Information (**Tables D-H in** **S1 Appendix**).

## 4. Real-data application

### 4.1. Data description

Participants in this study were drawn from ADNI, a long-term, multicenter longitudinal study designed to identify clinical, genetic, and biochemical markers associated with AD progression. ADNI-1 was launched in 2003 and enrolled individuals aged 55–90 years from 63 sites across the United States and Canada; subsequent phases (ADNI-GO, ADNI-2, and ADNI-3) followed existing participants and recruited additional cohorts. Details of the ADNI study design have been described elsewhere [22–24]. The ADNI database provides access to demographic information, questionnaire data, cerebrospinal fluid (CSF) biomarkers, and multi-omics measurements including DNA methylation (DNAm).

We examined baseline demographic and genetic covariates and focused on baseline CSF ABeta42 status as the primary exposure. Following Hansson et al. [25], who derived Elecsys assay-specific ABeta42 cut points with high concordance to amyloid PET and validated them in ADNI, we used 977 pg/mL as the threshold. Participants were classified into low (≤977 pg/mL) and high (>977 pg/mL) ABeta42 groups, with status treated as a binary exposure. We then estimated the causal effect of baseline ABeta42 on AD severity at 12 months, measured by ADAS-11, which ranges from 0 to 70, with higher scores indicating more severe cognitive impairment.

Prior studies have shown that DNAm alterations differ systematically between cognitively normal individuals and AD patients and are associated with CSF biomarkers including ABeta42, suggesting that amyloid-related processes in the central nervous system are at least partly reflected in the peripheral epigenome [9]. Accordingly, to mitigate potential confounding, we included baseline genome-wide peripheral blood DNAm markers as candidate covariates and applied a standardized preprocessing pipeline: (i) excluding probes with detection P-values > 0.05; (ii) removing sex-chromosome and sex-related probes; (iii) discarding probes harboring single nucleotide polymorphisms (SNPs) at the CpG site; (iv) removing cross-reactive probes; and (v) averaging DNAm levels across repeated measurements for the same individual. After quality control, 865,859 CpG sites, age, gender, and education were retained. Given the extreme covariate dimensionality, we further conducted an epigenome-wide association study (EWAS) and selected the top 100 CpG sites ranked by Bonferroni-adjusted P-values to serve as the covariate set in the subsequent causal analysis.

The final analytical sample comprised 1,269 eligible participants, including 49 African American and 1,220 non-Hispanic White individuals. In the analysis, African Americans were defined as the target domain with a limited sample size, whereas non-Hispanic Whites were defined as the source domain with a substantially larger sample size. All participants had complete exposure and outcome data. For partially missing covariates, we applied chained-equation imputation with predictive mean matching, implemented with the mice package, to generate one completed analytic dataset. Building on our simulation results demonstrating the limited applicability and instability of conventional estimators, we compared Lasso, DML, TMLE, $l_{\text{1}}$-TCL, and DSTCL methods. After adjusting for age, sex, years of education, and the selected CpG sites, we estimated the causal effect of interest and its 95% confidence interval. The confidence intervals were obtained via bootstrap with 500 resamples.

### 4.2. Results

Table 2 shows that the source and target domains were characterized using the same measured covariates, although their covariate distributions were not identical. African American participants were slightly younger and more often female, and they tended to have higher ABeta42 levels. We further assessed source-target comparability using standardized mean differences and distribution comparison tests for demographic, clinical, and selected DNAm covariates. These diagnostics showed that age was well balanced and education was acceptably balanced, whereas sex, ABeta42 status, and a subset of selected CpG covariates showed moderate source-target differences (**Table I in** **S1 Appendix**).

**Table 2:** Characteristics of African American and Non-Hispanic White participants.

	African American (n = 49)	Non-Hispanic White (n = 1,220)	P value
**Age, median (IQR)**	62.0 (60.0-66.0)	63.0 (58.0-67.0)	0.744
**Gender, %**			0.009
**Male**	18 (36.7)	693 (56.8)	
**Female**	31 (63.3)	527 (43.2)	
**Education, median (IQR)**	16.0 (14.0-18.0)	16.0 (14.0-18.0)	0.189
**ABeta42, median (IQR)**	1001.0 (565.6-1605.0)	843.2 (594.2-1396.8)	0.568
**ADAS-11, median (IQR)**	7.0 (4.3-10.0)	9.0 (5.3-14.3)	0.025

**Fig 7:**
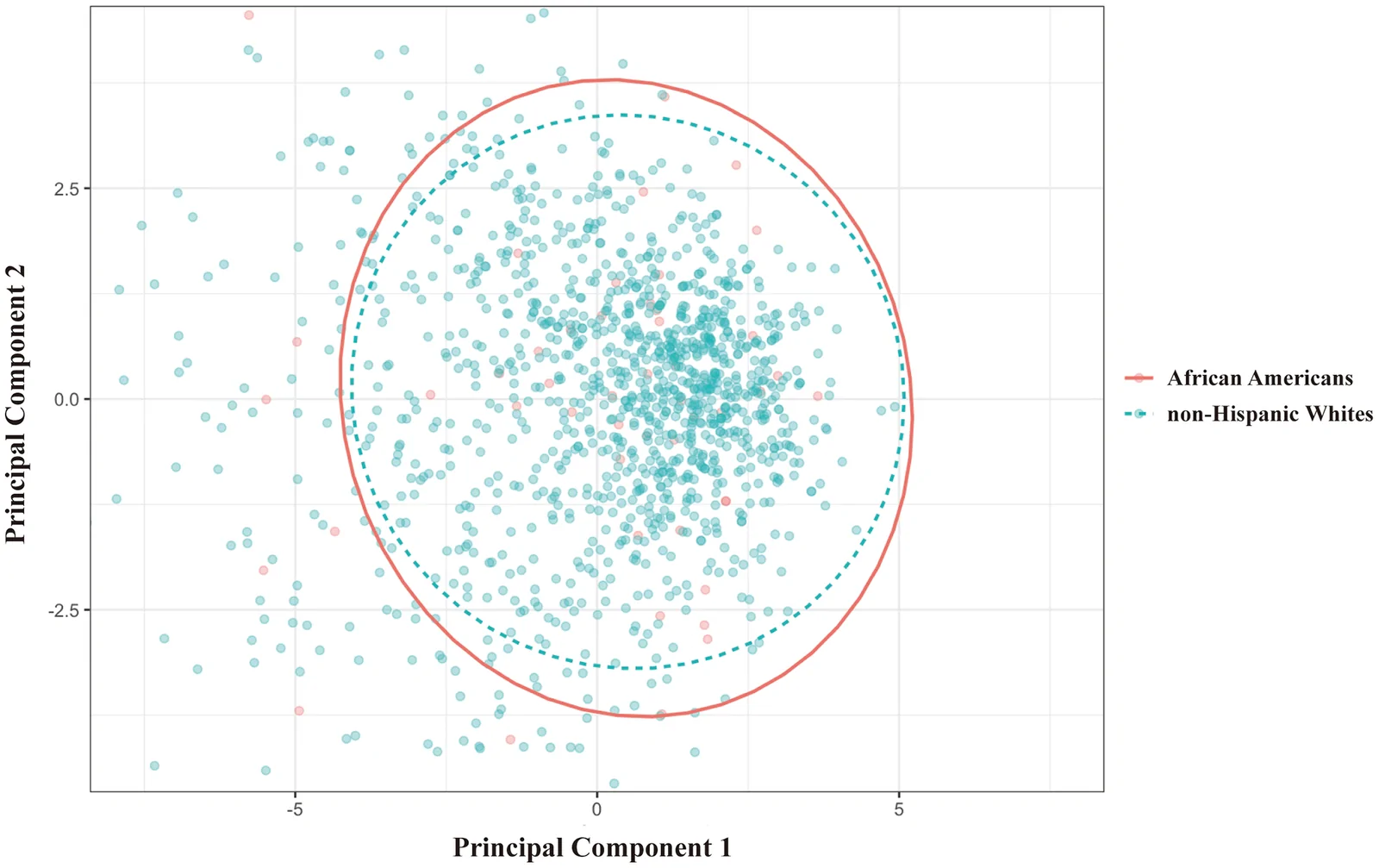
Results of principal component analysis of top 100 CpG sites in two populations.

Consistent with the observed source-target comparability in measured covariates, principal component analysis of the CpG sites demonstrated substantial overlap between African American and non-Hispanic White participants in the PC1-PC2 space, with closely aligned group centers and largely overlapping confidence ellipses (Fig 7). This pattern indicates the presence of a shared global structure in the DNAm feature space used for modeling, despite potential population-specific variation at individual loci. Taken together, these diagnostics suggest that the two domains were not identical but shared a broadly comparable measured covariate space. Therefore, transfer learning was considered plausible in this application, while the results should be interpreted together with these source-target distributional diagnostics.

To evaluate the stability of the real-data DSTCL estimate in the small target sample, we examined propensity-score overlap and effective-sample-size diagnostics. In the primary analysis, the propensity scores in the target domain ranged from 0.30 to 0.52, with no values below 0.05 or above 0.95. The effective sample size was 46.78 out of 49, suggesting good overlap between exposure groups and limited instability from inverse probability weighting (**Table J in** S1 Appendix).

As shown in Table 3, all methods yielded broadly similar negative estimates of the effect of high versus low baseline ABeta42 status on ADAS-11 scores in African Americans under the stated causal assumptions. As an imputation sensitivity analysis, we repeated the DSTCL analysis using a simple single-imputation strategy, with median imputation for continuous variables and mode imputation for categorical variables. The estimate remained negative, consistent with the primary analysis, although the bootstrap 95% CI was wider under simple imputation, suggesting reduced stability with the simpler imputation strategy (Table K in S1 Appendix). Lower ABeta42 levels indicate greater amyloid burden. Therefore, these negative estimates should be interpreted according to the ABeta42 coding, such that lower ABeta42, corresponding to higher amyloid burden, is associated with higher ADAS-11 scores and worse cognitive performance. Furthermore, the target-only Lasso produced wide confidence intervals, reflecting substantial variability in the small target sample. By contrast, the other methods yielded more stable estimates with 95% confidence intervals that exclude the null, and the proposed DSTCL estimator achieved the narrowest 95% CI, indicating the highest precision among all approaches.

**Table 3:** Causal effect estimates and 95% CIs among African American participants.

Methods	Estimate	95% CI
**Lasso**	-5.02	(-11.23, -0.50)
**DML1**	-7.56	(-12.29, -3.46)
**DML2**	-8.12	(-11.94, -3.49)
**TMLE**	-6.05	(-15.32, -2.15)
$l_{1}$ **-TCL**	-5.24	(-9.13, -1.31)
**DSTCL**	-6.33	(-9.59, -2.48)

## 5. Discussion

In this study, we proposed a double shrinkage transfer causal learning method for causal effect estimation. Compared with existing approaches, DSTCL improves performance by simultaneously regularizing the initial source-domain estimation and the source-target difference parameters, thereby improving the accuracy and stability of parameter estimates in the target domain and yielding more reliable and stable causal effect estimates.

Through extensive simulation studies covering a range of data generating mechanisms, DSTCL achieved finite-sample performance close to that of an ideal oracle estimator with prior knowledge. Compared with other methods, DSTCL generally yielded the smallest Bias and MSE, together with relatively high coverage. In the real-data application, we applied DSTCL to the ADNI dataset to estimate the causal effect of baseline CSF ABeta42 status on 12-month ADAS-11 cognitive scores among African Americans, adjusting for age, sex, years of education, and a set of DNAm covariates. DSTCL and the compared methods consistently suggested that lower baseline CSF ABeta42, reflecting greater cerebral amyloid burden, was associated with worse 12-month cognitive performance among African Americans, supporting a positive relationship between amyloid pathology and cognitive impairment. Meanwhile, DSTCL produced the narrowest 95% confidence interval among all methods, indicating high statistical precision. From a biological perspective, reduced CSF ABeta42 levels typically indicate increased cerebral amyloid plaque deposition, which in turn triggers a cascade of downstream pathological changes and ultimately leads to progressive impairment of multiple cognitive domains, including memory, attention, and executive function.

Several limitations should be noted. Firstly, DSTCL relies on a sparse parameter-shift assumption, under which only a limited subset of model parameters differ between source and target domains. While this assumption appears reasonable in our setting, it may not hold in applications involving markedly different diseases or highly heterogeneous populations, and future work should explore more flexible notions of cross-domain similarity and corresponding penalty structures. In addition, because DSTCL applies *l*_1_ regularization in both the source-domain estimation and source-target correction steps, double shrinkage may introduce a bias-variance trade-off; we mitigated this risk through cross-validation and regularization sensitivity analyses. Secondly, although DSTCL does not require identical marginal covariate distributions across domains, its performance still depends on sufficient source-target covariate overlap and the plausibility of sparse parameter differences. In the ADNI application, distributional diagnostics indicated both shared structure and measurable differences between African American and non-Hispanic White participants. Moreover, because the target domain included only 49 African American participants, finite-sample instability and overfitting cannot be completely ruled out, despite favorable propensity-score overlap and effective-sample-size diagnostics. Thirdly, establishing a complete formal theory for DSTCL remains challenging because the method combines transfer learning, two *l*_1_-regularized steps, and a doubly robust estimating equation. Existing studies suggest that such theory requires source-target similarity, sparsity, and careful control of regularized nuisance estimation errors [15,26,27]. Formal consistency and asymptotic normality results remain an important direction for future work. Finally, the current study focused on binary exposure and continuous outcomes and used an EWAS-based screening step to select CpG sites, which may not fully preserve the methylome’s correlation structure. Future work should extend DSTCL to more general exposure and outcome types and explore alternative dimension-reduction strategies. Because the ADNI application was based on observational data, the estimated effects should also be interpreted cautiously and conditional on standard causal assumptions, as residual confounding may remain despite adjustment for measured covariates. Notwithstanding these limitations, our results indicate that DSTCL offers a practical and robust approach for causal effect estimation in biomedical studies.

In conclusion, DSTCL provides a stable transfer causal learning framework for causal effect estimation when the target population has a limited sample size. The simulation studies showed favorable finite-sample performance, and the ADNI application suggested that lower baseline CSF ABeta42, reflecting greater amyloid burden, was associated with worse 12-month cognitive performance among African American participants.

## Supporting information

S1 Appendix Supplementary simulation and real-data analyses. This appendix contains supplementary simulation methods, main simulation results, sensitivity analyses, computational diagnostics, and supplementary analyses of the ADNI data, including the following tables: Table A. Results across methods as the source-domain sample size varies (Scenario 1). Table B. Results across methods as h-transferability varies (Scenario 2). Table C. Results across methods as the covariate dimension varies (Scenario 3). Table D. Regularization sensitivity analysis for DSTCL. Table E. Sensitivity analysis under increasingly extreme true propensity scores. Table F. Propensity-score trimming sensitivity analysis for DSTCL. Table G. Covariate-shift sensitivity analysis for DSTCL. Table H. Computational time and convergence diagnostics for DSTCL as the covariate dimension increases. Table I. Variable-level source-target distribution diagnostics in the ADNI application. Table J. Propensity-score and effective-sample-size diagnostics in the ADNI analyses. Table K. DSTCL estimates from the ADNI imputation analyses.
(DOCX)
